# Knockdown of *SLC38* Transporter Ortholog – *CG13743* Reveals a Metabolic Relevance in *Drosophila*

**DOI:** 10.3389/fphys.2019.01592

**Published:** 2020-01-21

**Authors:** Tanya Aggarwal, Sourabh Patil, Mikaela Ceder, Maher Hayder, Robert Fredriksson

**Affiliations:** Molecular Neuropharmacology, Department of Pharmaceutical Biosciences, Uppsala University, Uppsala, Sweden

**Keywords:** solute carrier, *SLC38*, CG13743, *Drosophila*, metabolism, behavior, transporter

## Abstract

Solute Carrier (SLC) is a cluster of families of membrane bound transporters, of which many members lack defined substrate profile, and many more are poorly characterized. Many play a vital role in regulating metabolic systems, protein synthesis, and post translational modifications. *SLC38* is one of the families of SLCs, which are also known as sodium-coupled neutral amino acid transporters (SNATs). In mice, it has 11 members (SNAT1-11) but in *Drosophila* there are two homologs for the *SLC38* family; *CG13743* and *CG30394.* Here, we show characteristics of *Drosophila CG13743* which closely resembles SLC38A11 in humans. SLC38A11 still remains an orphan member of the SLC38 family which has not been functionally well studied. We used the *UAS-GAL4* system to investigate and control gene expression using RNAi lines for ubiquitous knockdown of the *CG13743* gene. It was found to be expressed mainly in salivary gland and brain. Knockdown flies had reduced body weight and consumed less sugar compared with controls. The gene knockdown also affected stored energy pools (lipids and glycogen) and influenced feeding pattern and total activity. In all, this shows novel findings for the characterization of *CG13743* in *Drosophila* and a possible role in maintaining general metabolic pathways and behavior of the fly.

## Introduction

Solute carriers (SLC) are known to be the largest group of secondary transporters with 456 members divided into 65 families in human (Perland and Fredriksson, 2017). These are transmembrane transporter proteins regulating uptake and excretion, transport and transfer of amino acids, neurotransmitters, sugars, and drug molecules across membranes (Rask-Andersen et al., 2013). They are also responsible for controlling homeostasis in the body, and also serve as drug targets (Hediger et al., 2013). Disturbance or obstruction in the SLCs encoding genes have been linked to various pathological disorders such as inflammatory bowel disease, jaundice, rotor syndrome, and hyperbilirubinemia (Lin et al., 2015).

The solute carrier family 38 (*SLC38*) is an amino acid transporter family consisting of 11 members termed as *SLC38A1–SLC38A11* (Bröer, 2014). They are also referred to as sodium-coupled neutral amino acid transporters (SNATs). Members of *SLC38* family are membrane transporters found in all cell types. They mediate transport of Na^+^-dependent net uptake and efflux of amino acids and play an important role in the Glutamate/GABA-glutamine cycle working between neurons and astrocytes (Bröer, 2014). Despite their transport function, they are also suggested to act as transceptors (Hundal and Taylor, 2009). Chan et al. (2016) studied the importance of *SLC38A3* and its capacity to transport glutamine and was shown to have a critical role in amino acid metabolism. Several other studies have pointed toward varied roles of *SLC38* family members in metabolism, homeostasis, and neurotransmission (Bagchi et al., 2014; Hägglund et al., 2015; Hellsten et al., 2018).

The physiological role of many *SLC38* members and their implications in disorders is not completely known. In this paper, we report their role in the model *Drosophila melanogaster*, which offers balance between possibilities for genetic manipulation and similarity to the human genome (Featherstone, 2011). In *Drosophila*, phylogenetic analysis revealed that there are two homologs for *SLC38*; one possible orthologous to *SLC38*A10 (*CG303094*) and one that are ancestral to the rest of the *SLC38* members (*CG13743*) (Sundberg et al., 2008). So far very little is known about these homologs in *Drosophila* and here we will focus on one of them – *CG13743*.

## Materials and Methods

### *Drosophila* Stocks and Maintenance

*CG13743-UAS* RNAi KK line was ordered from Vienna *Drosophila* Resource Centre (v110773), *CG13743-GAL4* line from Kyoto stock centre (103883), *w*^1118^ *(5905)* and *UAS-GFP* (6658) line were ordered from Bloomington stock centre and *da-GAL4* was a kind gift from Prof. Dick Nässel, Stockholm University. Fly stocks were maintained on Standard Jazz-mix food (Fisher Scientific), supplemented with yeast extract (Genesee Scientific), at 25°C and 60% humidity on a 12-h light, 12-h dark cycle. For gene localization, offspring were collected from a cross made between *CG13743-GAL4* > *Pin/Pin-UAS GFP*. For gene knockdown experiments, crosses were made between control lines (*da-GAL4* > *w*^1118^), RNAi control (*w^1118^* > *UAS-CG13743 RNAi*) and knockdown cross (*da-GAL4* > *UAS-CG13743 RNAi*). Male offspring were collected from crosses and aged for 5–7 days on normal food and further used for experiments.

### GFP Expression for Gene Expression Identification

To detect *CG13743* expression we used UAS-GAL4 system, in which flies are used with gene of interest tagged to GAL4 and crossed with *UAS-GFP* flies. The offspring then obtained are used to check the GFP expression at the site where gene of interest localizes. Here *CG13743-GAL4* flies were crossed with *UAS-GFP* flies. Expression was studied at both the important developmental stages – larval stage and adult stage. Larval stage consists of three phases – first instar, second instar, and third instar larva. Larvae were dissected in third instar stage where the organs like brain, SG, imaginal disks are distinguishable. Adult males were also dissected to check different regions of the body for expression. The collected tissue was either stained with Dapi (Thermofisher) or directly mounted in mowiol anti-fade media (25 g mowiol 4–88 in 100 mL 1×PBS, pH 8.0, 50 mL glycerol, 3 mL of 1% Thimerosal, and 100 μg/ml *n*-propyl gallate – Sigma-Aldrich) on slide. Images were obtained using Nikon Eclipse TE2000-U inverted microscope with a Nikon Digital Sight DS-5Mc camera.

### Phylogenetic Relationship

The sequences were aligned using T_Coffee (Waterhouse et al., 2009) with default settings and manually inspected in Jalview (Waterhouse et al., 2009) where the N- and C-termini was identified and removed. The resulting sequences were realigned using T_Coffee with default settings. The phylogenetic relationships between the sequences were inferred using the Bayesian approach as implemented in mrBayes 3.2.2 (Huelsenbeck and Ronquist, 2001; Ronquist et al., 2012) to obtain the tree. The analysis was run via the Beagle library (Ayres et al., 2012) on an NVIDA 980Ti graphics card on six chains (five heated and one cold) with two runs in parallel (*n* runs = 2) under the mixed amino acid model with eight gamma categories and invgamma as gamma rates for a total of 2,000,000 generations. The tree was plotted in FigTree^1^.

### Body Weight

We have used male flies for all experiments as for females, the hormone cycle can affect the activity and metabolism and may give inconsistent results. Adult male flies aged 5–7 days old were weighed by placing groups of five flies in a vial (six replicates of *N* = 5 were used per genotype). The vial was pre weighed and the weight was subtracted from total weight of the flies and the vial. And the average weight/fly was plotted.

### Phenotypic Analysis

Adult male flies aged 5–7 days old were anesthetized and visualized under the microscope. Images were taken on Leica M125 and processed as a montage in Image J.

### RNA Extraction

The phenol-chloroform method was used for RNA extraction from tissue samples (Chomzynski and Sacchi, 1987). Briefly, ten flies were homogenized with TRIzol (Invitrogen, United States) followed by addition of Chloroform (Sigma-Aldrich). Samples were centrifuged at 13000 rpm for 12 min at 4°C. The aqueous layer, containing RNA was collected and Isopropanol (Solveco AB, Sweden) was added to precipitate RNA at −20°C for 30 min. Samples were centrifuged at 13000 rpm for 12 min at 4°C, to collect the RNA pellets, which were then washed with 75% ethanol (Solveco AB, Sweden) to remove the organic impurities. RNA pellets were incubated at 37°C for 30 min with RNAse free water (Qiagen GmBH, Germany) and DNAse incubation buffer (Roche GmBH, Germany) and DNAse I (Roche GmBH, Germany). DNAse was deactivated by adding EDTA and incubating the samples at 75°C for 10 min. The RNA pellet was air dried and resuspended in RNase free water. The RNA concentration was measured using a Nano drop ND 1000 spectrophotometer (Saveen Werner). Ten replicates of *N* = 10 were used per genotype.

### cDNA Synthesis

cDNA was synthesized from RNA using High capacity RNA to cDNA kit (Applied Biosystems) by following manufactures instructions. The cDNA was stored at -20°C and used for qPCR.

### Quantitative RT-PCR (qPCR)

Primers for fly samples *CG13743* was used according to the following sequence. *CG13743* forward 5′-GAACATCCGACAAATCGGCG-3′, reverse 5′-CATGAACTG CAGCAGGGAGA-3′; reference housekeeping genes: Rp49 forward_dw 5′-CACACCAAATCTTACAAAATGTGTGA-3′, reverse 5′-AATCCGGCCTTGCACATG-3′; Rp11 forward 5′- CCATCGGTATCTATGGTCTGGA-3′, reverse 5′-CATCGTAT TTCTGCTGGAACC-3′; Actin42a forward 5′-ACAACACTTC CGCTCCTT-3′, reverse 5′-GAACACAATATGGTTTGCTT ATGC-3′.

qPCR master mix was made by calculating per well volumes as – 1.5 μl DreamTaq Buffer (Thermo scientific), 0.1 μl 20 mM dNTP, 0.025 μl forward and reverse primer (100 pmol/μl), 0.25 μl of SYBR Green (1:50000; Invitrogen) in TE buffer (pH 7.8), 0.5 μl Dimethyl sulfoxide (Sigma Aldrich), and 0.04 μl DreamTaq polymerase (5 U/μl, Thermo scientific). 2 μl cDNA (10 μg/μl) per reaction was used as template. The measurements were run on CFX384 and CFX96 real-time system detection instrument (Bio-Rad Laboratories) according to following parameters: 3 min at 95°C initial denaturation, followed by 50 cycles of 10 s at 95°C, 30 s at 55–61°C (optimal temperature for each primer pair) and 30 s at 72°C, followed by a melting curve (+0.5°C per cycle, 81 cycles at 10 s intervals, starting from 55°C). Each sample was run in triplicates. Negative controls were included on each plate. All experiments were repeated twice. Data analysis was done using Bio-Rad CFX Maestro software and significance was calculated using GraphPad Prism 5.

### Refeeding Experiment Using flyPAD

Feeding experiments were performed in a similar fashion as Itskov et al. (2014). Male offspring (*n* = 32/genotype/diet) were raised on *ad libitum* normal *Drosophila* food. Flies were individually transferred to the flyPAD (fly Proboscis and Activity Detector) behavioral arena by mouth aspiration and left to feed on either 5% sucrose (Sigma-Aldrich, Sweden), or 0.5% margarine (4.7% monounsaturated fat, 2.8% polyunsaturated fat, 1.9% saturated fat) (ICA, Sweden) or a 10% yeast solution (Sigma-Aldrich, Stockholm, Sweden) in 1% agarose (Sigma-Aldrich, Sweden). For each experiment, fresh aliquots of prepared food were melted at 70 °C in a heat block immediately before adding 4 μl to the arena for the experiment that continued for a period of 2 h. flyPAD data were recorded using the Bonsai framework and sips and activity bouts were calculated with MATLAB as described previously in Itskov et al. (2014) paper. Data obtained were compared between knockdown and control groups using GraphPad Prism 5.

### Activity Monitoring Using DAMS

*Drosophila* activity monitoring system (DAMS) was used to study activity pattern in a similar way as Chiu et al. (2010). The system records when a fly crosses the center of the locomotor tube to break the infrared beam passed across the middle (Dubowy and Sehgal, 2017). To determine the effect of *CG13743* knockdown on fly’s activity, 32 male flies from both controls and knockdown flies were used. 32 tubes per monitor, containing either normal food or starvation food (1% agarose with water) were prepared and one fly was placed inside each tube. Data was recorded for 4 days using DAMS. Flies were allowed to get accustomed to the tubes and the first 24 h reading was not considered as it took time for the flies to adjust to the new environment. Experiment was repeated twice with *n* = 32 flies/genotype. Data was analyzed using Sleep and Circadian Analysis MATLAB program (SCAMP) software. For locomotor activity on normal food, the data of 3 days was averaged in software to calculate the activity during 24 h. For starved flies, the data of first 24 h during starvation was used to calculate the activity since our aim was also to calculate starvation resistance for which we need data from the starting point. Further the outliers were eliminated using online grubs test tool and graphs were plotted in GraphPad Prism 5.

### Triacylglyceride (TAG) Assay

Five adult flies per replicate were homogenized in phosphate-buffered saline with 1% Triton-X (PBST) on ice. 10 μl of homogenized sample was removed and stored at −80 °C to be measured later for protein content. Samples were heat inactivated at 70 °C for 10 min. Heat-treated homogenates were incubated at 37 °C for 45 min in replicates with either triglyceride reagent (Sigma) or PBST. Samples were transferred to 96 well plates and Free Glycerol Reagent (Sigma) was added for 5 min at 37 °C. Absorbance was recorded using a multiscan microplate spectrophotometer (Multiskan GO; Thermo Fisher Scientific) 540 nm. Experiment was designed using six replicates with five flies/genotype/condition. TAG was determined by subtracting the amount of free glycerol in the PBST-treated sample from the total glycerol present in the sample treated with triglyceride reagent. Glycerol standard solution (Sigma) was used to make 0–1 mg/ml of triolein equivalent standards which was treated like the fly samples.

### Sugar Assay

Stored body Glycogen and trehalose as well as circulating glucose and trehalose concentrations were measured using Liquick Cor-Glucose diagnostic kit (Cormay, Poland). For each condition, eight biological replicates were used with each containing around 10 mg (10–12 flies) of decapitated male flies. Briefly, PBS was added to the flies and centrifuged at 3000 rpm for 6 min at 4°C to extract haemolymph. Haemolymph was used measure circulating trehalose and glucose. To extract body supernatant, the remaining bodies of the 10 flies were homogenized in PBS, and homogenates were centrifuged at 13000 rpm for 20 min at 4°C. The supernatants collected were incubated with porcine kidney trehalase (Sigma-Aldrich, Stockholm, Sweden) to digest trehalose to glucose at 37°C for 18–24 h. Glycogen digestion was achieved by adding amyloglycosidase from *Aspergillus niger* (Sigma) to body supernatant at 25°C for 18–24 h. Glucose levels from all substrates were measured with Liquick Cor-Glucose diagnostic kit according to manufactures instructions. Absorbance was measured at 500 nm for each replicate of each substrate on a multiscan microplate spectrophotometer (Multiskan GO; Thermo Fisher Scientific) and converted to a millimolar concentration of glucose using a linear regression obtained by a calibration curve made from serial dilution of a sample with a known glucose concentration. Glucose measurements were then converted back to the units of their original substrates. Experiment was done using ten replicates of 10–12 flies/genotype/condition.

### Data Analysis

Mean and SEM were calculated from all the replicates of each experiment. All analysis was performed with GraphPad Prism version 5. *T*-tests and one-way ANOVA analysis was performed with appropriate *post hoc* test for multiple comparisons for each experiment as mentioned in figure legends.

## Results

### *CG13743* Clusters With Human *SLC38* and Is Expressed in Salivary Gland and Brain

The phylogenetic relationship was studied using sequences from mouse and *Drosophila* to identify for the ortholog for *CG13743*. The phylogenetic tree shows that *CG13743* clusters closely with human SLC38 family and in close proximity to *SLC38A11* (Supplementary Figure S1D). *SLC38A11* is an orphan transporter from SLC38 family that has not been studied extensively and lack characterization and functional relevance in mouse model (Hellsten et al., 2018). Therefore, in this paper we have used *Drosophila* to show the functional relevance of *CG13743.*

**FIGURE 1:**
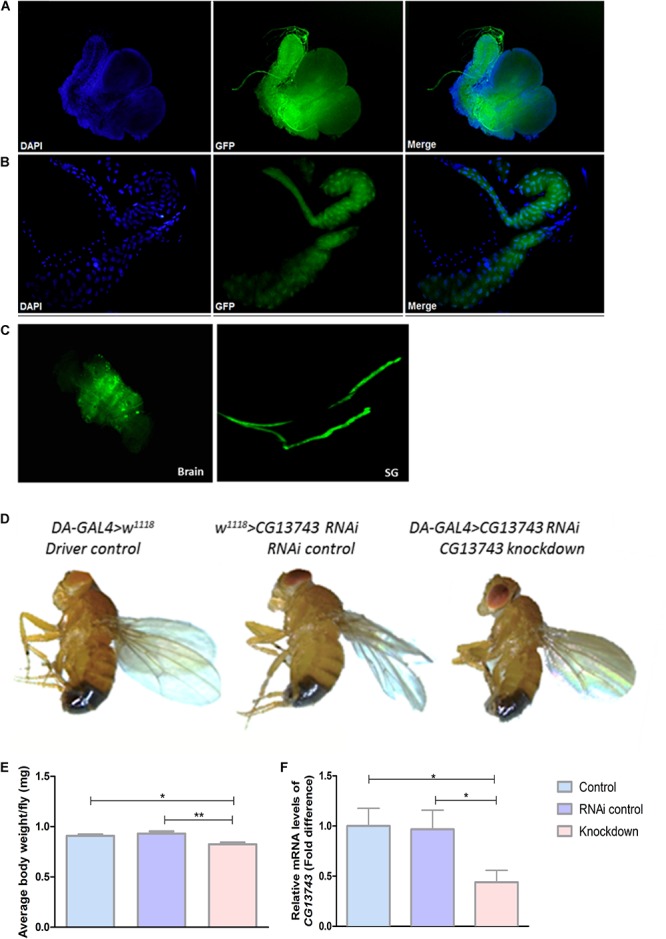
*CG13743* expression, phenotype, and knockdown. *CG13743* expression was visualized by crossing *UAS-GFP* with *CG13743-GAL4* flies. Larvae and adult were dissected to stain with DAPI and GFP to check for *CG13743* expression. *CG13743* was found to be expressed mainly in **(A)** brain and **(B)** salivary gland (SG) in larva and also in **(C)** adults – where GFP (green) shows localization of *CG13743* expression, DAPI (blue) indicates cell nuclei, Merge shows overlap of GFP and DAPI, *n* = 10; *CG13743* expression was reduced ubiquitously using *DA-GAL4* with *UAS-CG13743 RNAi* flies. Phenotype was assessed by capturing the morphology of knockdown flies (*DA-GAL4* > *UAS-CG13743 RNAi*) compared to driver control (*DA-GAL4* > *w*^1118^) and RNAi control (*w^1118^* > *UAS-CG13743 RNAi*) by microscope. **(D)** No body size difference was observed between the three groups pf flies. Body weight was measured for all the three groups of adult flies. **(E)** *CG13743* knockdown flies show reduced body weight compared to both the controls; *n* = 25 per genotype, one-way ANOVA with Kruskal–Wallis and Dunn’s *post hoc* test for multiple correction was performed (^∗^*p* < 0.05, ^∗∗^*p* < 0.01). Error bars denote ± SEM. *CG13743* knockdown was verified using qPCR. **(F)** The expression of *CG13743* was reduced compared to both controls. Normalization was performed against three housekeeping genes and relative mRNA expression levels were plotted compared with the both controls, and driver control (blue) was set to 1. Gene expression difference was calculated using one-way ANOVA and bonferroni’s multiple correction (^∗^*p* < 0.05). Error bars denote ± SEM.

The gene expression of *CG13743 in vivo* was studied using GAL4-UAS system coupled to GFP. The expression was identified using offspring obtained by crossing *CG13743-GAL4* flies with *UAS-GFP* flies. Expression was mainly found in salivary gland (SG) and brain in larval stage (Figures 1A,B). Expression of *CG13743* was also investigated in adult flies (Figure 1C) where similar expression patterns were found. We also examined other parts of the larval body and no GFP expression was found with control DAPI shown in blue in the larval body (Supplementary Figures S1A–C).

### Knockdown of *CG13743* by RNAi

*CG13743* knockdown flies were used for all further experiments. *CG13743* gene expression was reduced in the fly using *DA-GAL4* (*daughterless-GAL4*), a transgenic transposon with ubiquitous expression in the organism (Cronmiller and Cummings, 1993) and crossed it with *CG13743-UAS-RNAi* line. The offspring (knockdown flies; *DA-GAL4* > *UAS-CG13743 RNAi*) obtained from the cross were verified for gene knockdown with qPCR and expression was compared with appropriate controls (*DA-GAL4* > *w^1118^, w^1118^* > *UAS-CG13743 RNAi*). Relative mRNA transcript levels of *CG13743* were measured and knockdown was verified with expression reduction over 50% (Figure 1F).

### *CG13743* Knockdown Affects Body Weight

After verification, phenotypic analysis was done in terms of body size and body weight measurements. 5–7-day adult offspring were used from all the three groups. Body size was measured by capturing the images of knockdown and control flies using light microscopy. The body weight was measured by weighing multiple flies together and then taking the average weight per fly. We did not observe any differences in body size for knockdown flies compared with controls (Figure 1D). However, knockdown flies had reduced body weight compared with both control flies (Figure 1E). Knockdown flies had an average body weight of 0.75 mg whereas the controls had an approximate body weight of 0.95 mg.

### *CG13743* Knockdown Affects Feeding Behavior

**FIGURE 2:**
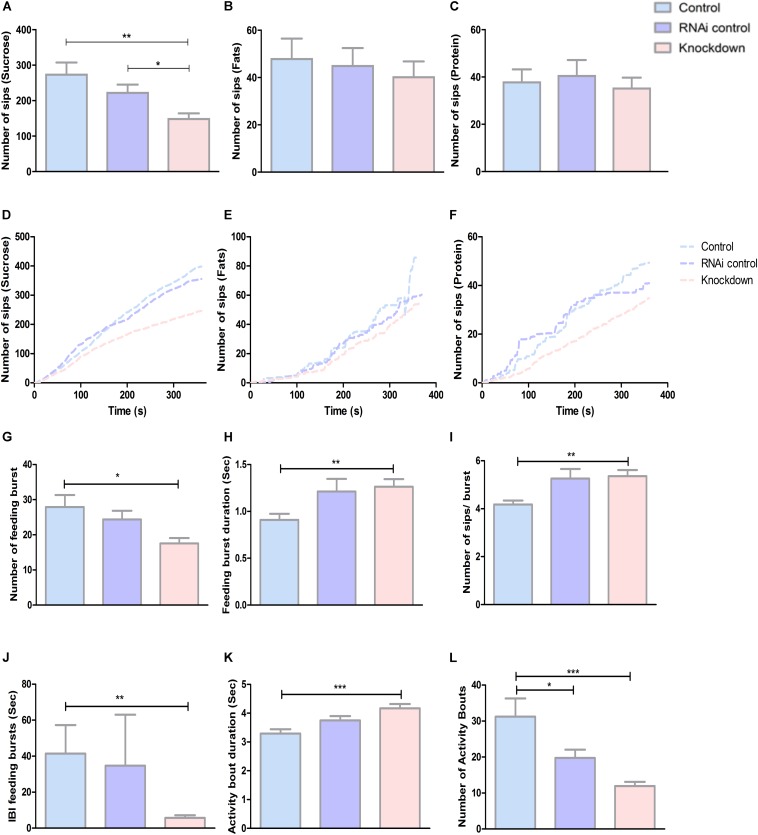
Food intake and feeding pattern. Food intake was measured by refeeding assay using flyPAD where flies were left to be fed on either sucrose or fats or protein. Number of sips was plotted which correlates to food intake in **(A)** Sucrose – Knockdown flies (*DA-GAL4* > *UAS-CG13743 RNAi*) show reduced sucrose intake compared to driver control (*DA-GAL4* > *w*^1118^) and RNAi control (*w^1118^* > *UAS-CG13743 RNAi*). **(B)** Fats – No difference was observed for fat intake. **(C)** Protein – No difference was observed for protein intake; Number of sips over time was also measured for **(D)** Sucrose, **(E)** Fats, **(F)** Protein and results show reduced food intake for knockdown flies in all the diets. Feeding pattern for sucrose diet was assessed using – **(G)** number of feeding burst, **(H)** feeding burst duration, **(I)** number of sips/bursts, **(J)** IBI feeding bursts, **(K)** activity bout duration, **(L)** number of activity bouts. *n* = 32/genotype per diet, one-way ANOVA with Kruskal–Wallis and Dunn’s *post hoc* test for multiple correction was performed (^∗^*p* < 0.05, ^∗∗^*p* < 0.01, ^∗∗∗^*p* < 0.001). Error bars denote ± SEM.

Studies have found that *SLC38* members are important for metabolic processes (Bröer, 2014). To check this further, we studied the result of *CG13743* knockdown on metabolism by investigating feeding behavior, food intake and macronutrient levels. Feeding behavior was observed using flyPAD (fly Proboscis and Activity Detector) which uses capacitive-based measurements to identify interaction of each fly with food (Itskov et al., 2014). The basic macronutrients essential in a standard diet (protein, fat, and carbohydrates) were measured to investigate food preference and intake. Flies were fed 5% sucrose (carbohydrates), 0.5% margarine (fats), or 10% yeast (protein) solution extract. The numbers of sips were assessed which refers to the total interaction of proboscis with food, which is proportional to food intake. Results show 35% reduction in number of sips on sucrose diet for knockdown flies compared with controls (Figure 2A). However, no difference was observed between knockdown and control flies for fat and protein diet (Figures 2B,C). The reduction in number of sips directly correlates to less consumption of food for knockdown flies on sucrose diet. And compared with the total number of sips for each diet, it was shown that flies prefer sucrose diet (Figures 2A–C). Also, when we investigated food preference over time, we found similar reduction for knockdown flies in all the three diets (Figures 2D–F). Investigating more in detail their feeding patterns, we analyzed feeding bursts which was defined as three or more consecutive sips separated by smaller inter-burst intervals (IBIs) and activity bouts that represents how regularly an animal accesses the food according to the previous study of Itskov et al. (2014). We found that number of feeding bursts and number of activity bouts was reduced for knockdown flies (Figures 2G,L). The feeding burst duration, activity bout duration, and sips per bursts were increased (Figures 2H–K). This suggests that *CG13743* knockdown affects food intake (in particular carbohydrates) and knockdown flies show altered feeding pattern compared to controls in terms of feeding burst and activity bouts.

### *CG13743* Knockdown Affects Stored Energy Molecules

**FIGURE 3:**
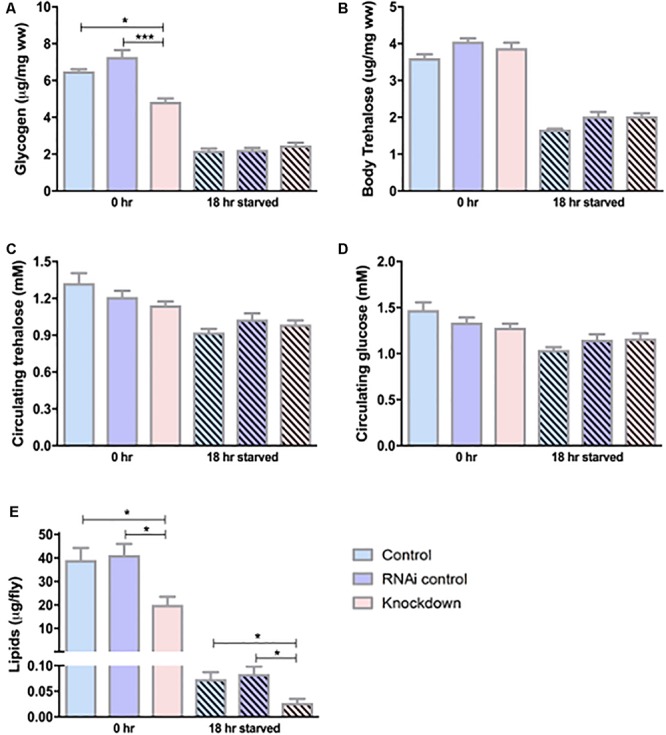
Carbohydrate and lipid levels. Circulating and stored levels of carbohydrates were measured using sugar assay kit. **(A)** Glycogen – knockdown flies show reduced glycogen under normal conditions, **(B)** Body trehalose – No difference was observed, **(C)** Circulating glucose – No difference was found, **(D)** Circulating trehalose – No difference was found; *n* = 100 per genotype per condition, one-way ANOVA with Kruskal–Wallis and Dunn’s *post hoc* test for multiple comparisons was performed (^∗^*p* < 0.05, ^∗∗∗^*p* < 0.001). Error bars denote ± SEM. Lipid levels were measured using TAG assay for adult flies under normal conditions (0 h) and starved conditions (18 h). *n* = 30 per genotype per condition, one-way ANOVA with Kruskal–Wallis and Dunn’s *post hoc* test for multiple comparisons was performed (^∗^*p* < 0.05). Error bars denote ± SEM. **(E)** Knockdown flies show reduced lipid levels at normal and starved conditions compared to controls.

From the above results, we show that *CG13743* is expressed in salivary gland and brain, and *CG13743* knockdown affects body weight and food intake in flies. We continued to study *CG13743* knockdown affects and the overall metabolic homeostasis. To examine this, levels of various molecules that are responsible for energy production were analyzed. Stored energy pools (Lipids, glycogen, body trehalose) from whole body extracts and circulating molecules (Glucose and trehalose) using haemolymph of adult flies at 0 h (normal conditions) and 18 h (after starvation for 18 h on agarose and water) were used for measurement. The TAG assay was performed to measure total lipid content and Liquick Cor-Glucose diagnostic kit to measure circulating and stored sugars. We found that knockdown flies had lower levels of lipids (50% lower at 0 h and 18 h) and glycogen (30% lower at 0 h) relative to body mass when compared with controls (Figures 3A, E). No differences were observed for glycogen at 18 h starvation and for body trehalose at both time points (Figures 3A, B). For circulating sugars, there was no difference observed between knockdown and controls at 0 or 18 h starvation (Figures 3C, D). In normal conditions, the above three storage molecules (Lipids, Glycogen and body Trehalose) serve as the biggest readily available energy resource for the fly (Tennessen et al., 2014; Matsuda et al., 2015). Low storage levels here denote that under condition of starvation, the knockdown flies might not have enough storage backup of energy pools which can in turn affect the activity and survival of flies.

### *CG13743* Knockdown Affects Total Activity

**FIGURE 4:**
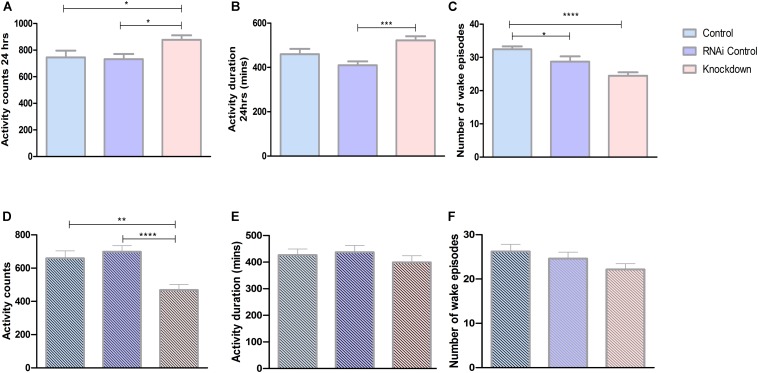
Activity behavior. Activity was analyzed using DAMS. All the three groups of flies were used to study behavior under normal conditions and starved conditions (food with agarose and water). Each fly was put in a small capillary tube with normal food or starved food and readings were recorded for 3 days. **(A)** Knockdown flies (*DA-GAL4* > *UAS-CG13743 RNAi*) show increased activity counts compared to driver control (*DA-GAL4* > *w*^1118^) and RNAi control (*w^1118^* > *UAS-CG13743 RNAi*). **(B)** Increased activity duration for knockdown flies, **(C)** reduced wake episodes for knockdown flies. Under starved conditions, knockdown flies show around **(D)** reduced activity counts, and no difference in **(E)** activity duration and **(F)** wake episodes. *N* = 64/genotype, one-way ANOVA with Kruskal–Wallis and Dunn’s *post hoc* test for multiple comparisons was performed (^∗^*p* < 0.05, ^∗∗^*p* < 0.01, ^∗∗∗^*p* < 0.001, ^*⁣*⁣**^p < 0.0005). Error bars denote ± SEM.

Our present results show that *CG13743* knockdown affects body weight, food intake as well as lipid and glycogen levels. To further explore the influence of *CG13743* knockdown on metabolic homeostasis, behavior assays to analyze total activity were performed. *Drosophila* activity monitoring system (DAMS) was used for a period of 3 days with normal food and under starved conditions (with only agarose and water) during 5–7 day for adult control and knockdown flies. Average activity behavior and wake episodes were assessed. Activity counts refer to the number of times the fly interrupts the infrared beam and is proportional to active state. Wake episode refer to the number of times the fly transits from immobile state (inactive for more than 5 min) to active state. Under normal conditions, we found that *CG13743* knockdown flies were more active with 17% more activity counts and higher activity duration time compared with both controls in normal conditions (Figures 4A,B). Knockdown flies showed a decrease in number of wake episodes which can suggest that they are active during longer periods of time at stretch (Figure 4C). The total period was also divided between 12 h dark and 12 h light period to check their behavior. We found that knockdown flies, specifically under the dark period, showed increased activity duration and mean activity duration but decreased wake episode number (Supplementary Figure S2)

On the other hand, during initial 24 h of starvation, *CG13743* knockdown flies had around 30% reduced activity counts compared with controls (Figure 4D). Whereas no changes were observed in the activity duration and number of wake episodes for knockdown flies compared to controls (Figures 4E,F). Comparing normal and starved conditions, knockdown flies showed a drop of almost 50% of total activity counts under starvation compared to normal conditions (Figures 4A,D). These findings show that *CG13743* is important for activity under normal and starved conditions.

## Discussion

*Drosophila* has similar organ systems as vertebrate counterparts which carry out basically the same metabolic functions (Leopold and Perrimon, 2007). Based on phylogenetic analysis, *CG13743* clusters closely to human *SLC38* family and in close proximity with *SLC38A11* (Supplementary Figure S1D). *SLC38A11* still remains understudied and is an orphan member in SLC38 family in vertebrates (Hellsten et al., 2018). *CG13743* clusters with human *SLC38A11* as an outgroup to the entire *SLC38A1-A6* cluster, suggesting it represents a pro-ortholog to the human *SLC38A1-A6* and *SLC38A11* branch. The branch separating the human *SLC38A7* and A8 branch from *CG13743* is relatively short, and it can therefore not be excluded that *CG13743* represents an ortholog also to this branch. This study show novel insights into functional characterization of the orphan member *CG13743* in *Drosophila*.

*CG13743* is mainly expressed in SG and brain at both larval and adult stage (Figures 1A–C). SG is the largest secretory organ in *Drosophila* with a known function of producing secretory glue to help attach pupa to the substrate (Farkaš, 2016). In addition to this, SG are also associated with production and release of apocrine secretion (Farkaš, 2016). Apocrine secretion helps to transport and distribute a large variety of cytosolic, cytoskeletal, membranous, Golgi, nuclear, mitochondrial protein, and signaling molecules across the interface and extrinsic surroundings (Farkaš, 2016). SG are also known to produce digestive secretions and store reserve food during larval growth (HSU, 1948). It also carries out a wide range of biological processes such as signaling, transport, etc. (Farkaš et al., 2014). This shows an important function of SG in metabolic functions in the fly. Studies have also found that central nervous system in fly plays an important role in monitoring energy balance and feeding regulation depending on nutritional state intrinsically (Pool and Scott, 2014). For *CG13743* expression in brain, we know that in mammals, there are well-established phenomenon where the brain process nutritional status signals and consequently act on that information (Owusu-Ansah and Perrimon, 2014). Studies have shown that *Drosophila* also possess various inter-organ communicating elements, which are regulated by insulin producing cells located in brain (Owusu-Ansah and Perrimon, 2014). Like *CG13743* in *Drosophila*, there are other genes located in brain and SG that have been reported to play role in nutrient balance and feeding behavior. For example-*Gustatory Receptor 43a* (*GR43a*) functions as a fructose receptor in taste neurons and brain (Miyamoto et al., 2012). It acts as a nutrient sensor for fructose in haemolymph and consequently promotes or supresses feeding in flies (Miyamoto et al., 2012). The other example is *CrebA* and salivary gland secretion protein gene (Sgs-1) which is expressed in SG at larval stage (Roth et al., 1999; Abrams and Andrew, 2005). *CrebA* and *Sgs-1* have been shown to play role in secretory functions of SG (Roth et al., 1999; Abrams and Andrew, 2005). Thus, taking into knowledge the function of SG and brain and the expression pattern of genes involved, it suggests that *CG13743* as solute carrier transporter can have a role in sensing and/or regulating nutrition, food intake or other metabolic functions.

We performed a knockdown study using *UAS-CG13743 RNAi* line with *DA-GAL4* to ubiquitously reduce the gene expression in the fly. We used knockdown flies and two controls (Driver control, RNAi control) to investigate phenotypic changes. We did not observe any changes in body size (Figure 1D) but we found a decrease in body weight for knockdown flies (Figure 1E). This can be correlated to the study done previously where *SLC38A3* knockout mice were found to have decreased body weight and were smaller compared to controls, which was a result of altered amino acid homeostasis (Chan et al., 2016). This supports the notion that *CG13743* in SG and brain can have a role in maintaining body weight. This can further have role in other metabolic processes, nutritional uptake or feeding behavior of flies, and to further investigate the consequences and effect on homeostasis we performed feeding assay and other general metabolic tests.

Regulation of feeding behavior is an important feature for metabolic homeostasis. Studies have shown that there are specific central neurons that can directly sense distinct circulating macronutrients and can alter feeding patterns (Pool and Scott, 2014). Another study has also revealed that amino acid imbalance can cease the feeding in *Drosophila* (Bjordal et al., 2014). Here, we used flyPAD to detect feeding pattern for sucrose, fats, and proteins. We found no significant differences for fat and protein intake for knockdown and control flies. We found that knockdown flies have reduced intake of sucrose compared with both controls (Figure 2A). Also, comparing axis for the total number of sips, our results show that our male flies prefer sucrose diet compared to fats and protein diet. This is in accordance to previous studies where it was shown that control male flies had a preference for sucrose diet (Jacob, 2012). From our results, reduced number of sips, number of feeding bursts and number of activity bouts show that knockdown flies approach the food for less number of times and ingest less food (Figures 2A,G,J,L). But the feeding burst duration, activity bout duration, and sips per bursts for knockdown flies were increased, which suggest that they ingest more food at one time and highly reduced IBI feeding burst adds to this that they eat the food with vigor (Figures 2H,K). Thus, *CG13743* knockdown can impact food intake (mainly for sucrose) which can be a possible reason for the reduction in body weight for knockdown flies (Figure 1E). A study has shown that depletion of Kir channels in salivary gland alters the performance of the gland and reduced sugar feeding in *Drosophila* (Swale et al., 2017). The altered feeding pattern seen in knockdown flies can be a result of the knockdown of the *CG13743* gene which being expressed in salivary gland can affect its function and in turn can also impact other mechanisms responsible for proper feeding in flies.

Insects like *Drosophila* have to spend energy regularly, and when they are not feeding, they have to survive on stored reserves collected during normal conditions (Arrese and Soulages, 2010). *Drosophila* contains two main circulating carbohydrate molecules – glucose and trehalose. Glucose is a monosaccharide required for cell metabolism and trehalose is a disaccharide used in insects mainly for instant energy for flight and also as a stored sugar for non-feeding and fasting conditions (Matsuda et al., 2015). *Drosophila* contains stored energy pools mainly in form of glycogen and lipids and also in the form of trehalose (Broughton et al., 2005). More than 90% of the stored lipids are triglyceride (TAG). Glucose is stored as glycogen in a polymeric form that can easily be degraded when needed and used by other tissues in form of trehalose (Arrese and Soulages, 2010). Compared to glycogen, lipids contain higher caloric content per unit of weight and contributes twice the amount of water on oxidation (Arrese and Soulages, 2010). To investigate the effects on metabolic homeostasis, we measured the levels of stored and circulating macronutrients at normal (0 h) and starved (18 h) conditions in fly. Knockdown flies had lower levels of lipids at normal and starved conditions compared to respective controls at each condition (Figure 3E). Also, knockdown flies had lower levels of glycogen at normal conditions, although under starvation no difference was observed (Figure 3A). This suggests that it is possible that knockdown flies cannot store enough lipids from the beginning which might lead to lack of energy pools to obtain energy from when they starve. Also, the lower levels of glycogen indicate that they have problems with storage but no problem retrieving the glycogen to use it during starvation and thus we do not see difference in glycogen after starvation. For the circulating sugars and body trehalose, there was no significant difference observed between knockdown and control flies under normal and starved conditions (Figures 3B–D). This also needs to be further investigated in detail at different time points to find out if the problem is with storage or retrieving of nutrients. Also, it can be possible that the effect is small and modulated by some other known genes responsible for metabolic regulation. Like in mammals, studies show that lipid and sugar homeostasis are also regulated by the interplay between insulin producing cells (*Ilps*) and a adipokinetic hormone (*Akh*) (Lee and Park, 2004; Broughton et al., 2005). From our results, *CG13743* knockdown either does not impact circulating sugars directly or the effect is little which is not significant.

*Drosophila* has behaviors that resembles most of the behavioral characteristics of mammalian activity (Shaw et al., 2000). Locomotor activity in fly is divided into a light-dark cycle, where flies display activity peaks during dawn and dusk, and sometimes an additional afternoon peak in natural conditions (Dubowy and Sehgal, 2017). We found that knockdown flies had increased activity counts and duration of activity counts compared to controls under normal conditions (Figures 4A, B). Interestingly, the number of wake episodes is significantly reduced in knockdown flies (Figure 4C). This shows that the knockdown flies are more active and for a longer period of time compared to control flies. Also, knockdown flies show a higher number of activity episodes and activity duration in dark period which can be due to aging, internal genetic factors (such as disturbances in *angiotensin converting enzyme-ACE, activity-regulated cytoskeleton-associated protein gene – Arc)* as well as external environmental factors (temperature) which will be studied further in future studies (Ishimoto et al., 2012). Increase in locomotor activity along with decrease in sugar consumption may have resulted in lower energy storage in lipid form (which has been shown earlier). In addition, under starved conditions we found that knockdown flies have reduced activity counts compared with both the controls (Figure 4D). This can be due to lower lipid storage in the body. This needs to, as mentioned before, be studied in detail either this is due to faster depletion of stored energy pools faster under prolonged starvation conditions or a reduced capability to retrieve stored nutrients.

We can here speculate that *CG13743* is an amino acid transporter with preference for glutamine, arginine, and histidine (Hägglund et al., 2015), similar to its mammalian orthologs. It is know that loss of *SLC38A3* (member of SLC38 family) in mammals results in downregulation of the mammalian target of rapamycin (mTOR) pathway which under normal conditions couples reduced amino acid availability to altered growth and homeostasis (Gu et al., 2005; Chan et al., 2016). Similarly, TOR in *drosophila* (dTOR) has also been reported to regulate growth and homeostasis in response to amino acid and nutrient availability (Zhang et al., 2000). Additionally, the genes responsible for feeding regulation and metabolism are found in SG and brain (Partridge et al., 2011; Pool and Scott, 2014), which are the regions where *CG13743* is also expressed. Similar to other genes that regulate feeding, it is highly possible that *CG13743* can also regulate feeding and thus be responsible for the results we obtained. There is still a need to study in detail the mechanisms behind the characteristics identified here, such as if the increased activity just a result of more locomotor activity for food search or if other factors are also involved? Also, we need to study if its reduced activity for starved flies is due to less internal energy pool or other metabolic impairments?

To conclude, we have found that *CG13743* knockdown can affect body weight, food intake, altered levels of stored energy reserves as well as activity in *Drosophila*. The results conclude that *CG13743* has a role in overall metabolism in *Drosophila* and reduced expression of *CG13743* is linked to imbalance in the metabolic homeostasis.

This paper was a characterization-based approach where we have investigated and evaluated the general role of *CG13743* gene in the *Drosophila* model which has not been done before. This was not a mechanistic study but important steps to enable further studies in terms of altered metabolism in context to solute carriers. The results reveal metabolic effects of *CG13743* gene knockdown and further pave way to characterize and investigate the other member in this context.

## Data Availability Statement

All datasets generated for this study are included in the article/Supplementary Material.

## Author Contributions

TA designed experiments and project, prepared crosses and offspring required for experiments, provided experimental methods (RNA extraction, sugar, tag and protein assays, dissections, staining, cDNA synthesis, DAMS, imaging), drafted the manuscript, compiled and analyzed figures, and acquired the funding. SP took care of crosses and methods (body weight, qPCR, flyPAD) and drafted parts of methods. MC assisted in designing experiments and reviewed and edited the manuscript. MH assisted in dissection and staining. RF assisted in analysis and interpretation of results, reviewed the manuscript, and acquired the funding. All authors reviewed the manuscript.

## Conflict of Interest

The authors declare that the research was conducted in the absence of any commercial or financial relationships that could be construed as a potential conflict of interest.

## Footnotes

1 http://tree.bio.ed.ac.uk/software/figtree/
