# Supplementary material for: Knockdown of SLC38 Transporter Ortholog – CG13743 Reveals a Metabolic Relevance in Drosophila
Source: Front Physiol. 2020 Jan 21;10:1592. doi: 10.3389/fphys.2019.01592 (PMC6985444; doi:10.3389/fphys.2019.01592)
Supplement: Supplementary file 1 [file Table_1.docx]

Supplementary Material

Supplementary Figure 1


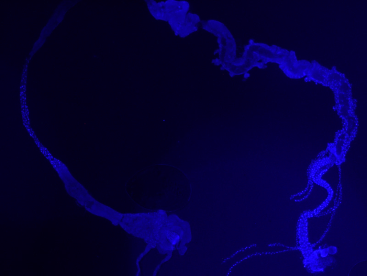


**DAPI**


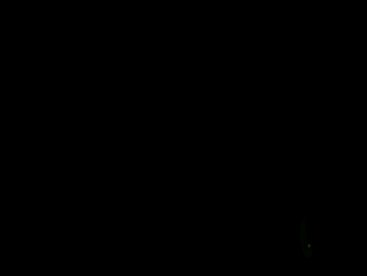


**GFP**

A

B


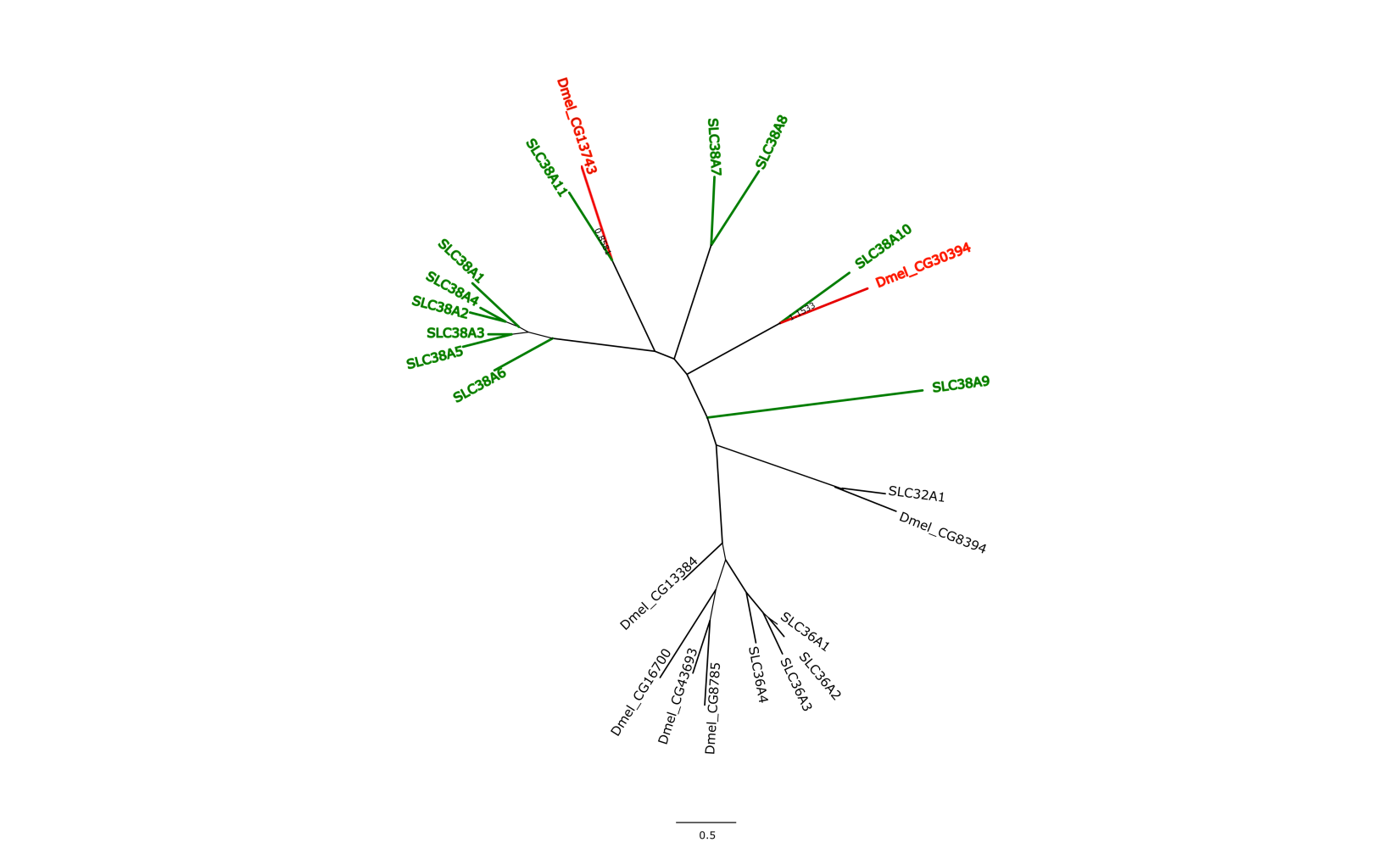


D


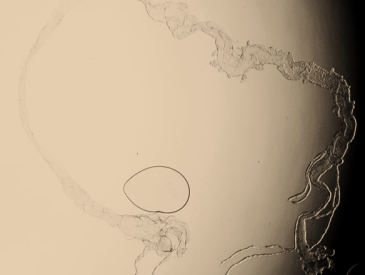


**BF**

C

**Supplementary Figure 1.** *CG13743* expression was visualised by crossing *Pin/Pin UAS-GFP* with *CG13743-GAL4* flies. Adult fly was dissected to check whole body for *CG13743* expression in adult fly. Staining of adult fly body in (A) Dapi and (B) GFP (C) Brightfield (BF). No GFP expression was found in rest of the body except brain and SG (Fig.1). (C) Unrooted phylogenetic tree as inferred using Bayesian phylogeny showing the phylogenetic relationship between human and *D. melanogaster SLC32*, *SLC36* and *SLC38* protein sequences. Human *SLC38* sequences are displayed in green and *D.melanogaster* *SLC38* sequences are displayed in red. Rest of the SLC members are denoted in black. *CG13743* clusters with human *SLC38A11* and to other members of SLC38 family.

## Supplementary Figure 2


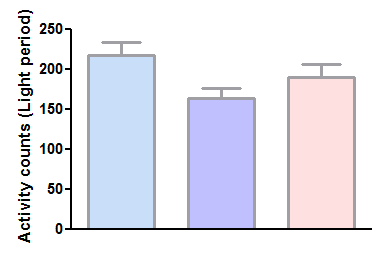

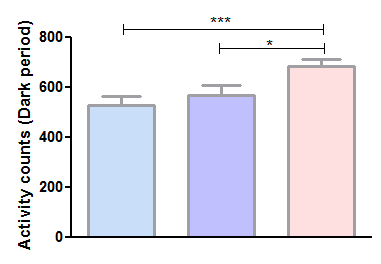

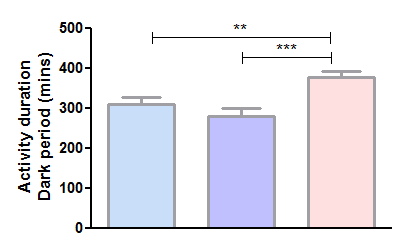

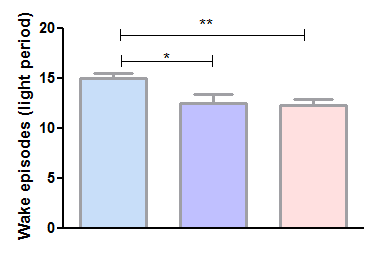

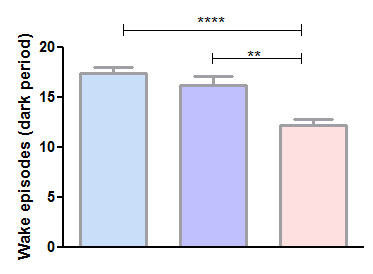

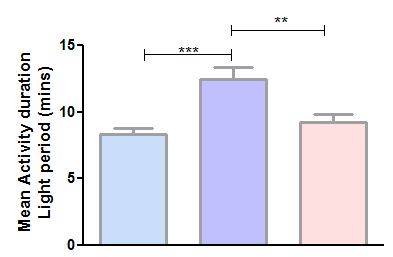

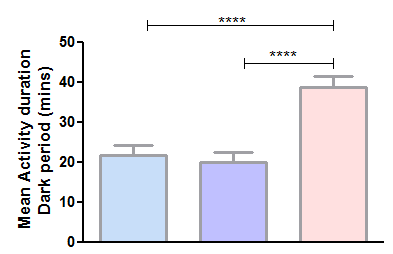

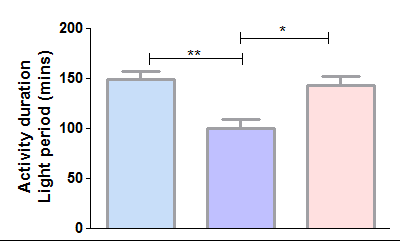


A

B

C

D

E

F

G

H

**Supplementary Figure 2**. Activity behaviour using DAMS. DAMS was used to record activity behaviour in knockdown and control flies in terms of A) activity counts- dark period, B) activity counts-light period, C) activity duration-dark period, D) activity duration-light period, E) number of wake episodes- dark period, F) number of wake episodes- light period, G) mean activity duration- dark peiod, H) mean activity duration- light period n=64/genotype, one-way ANOVA with Kruskal-Wallis and Dunn’s post-hoc test for multiple correction was performed (*p<0.05, **p<0.01, ***p<0.001, ***p<0.0005). Error bars denote ± SEM.
